# The Reduced Adaptability of H-Reflex Parameters to Postural Change With Deficiency of Foot Plantar Sensitivity

**DOI:** 10.3389/fphys.2022.890414

**Published:** 2022-06-29

**Authors:** Mengzi Sun, Kelsey Lewis, Jung Hun Choi, Fangtong Zhang, Feng Qu, Li Li

**Affiliations:** ^1^ Biomechanics Laboratory, Beijing Sport University, Beijing, China; ^2^ Department of Health Sciences and Kinesiology, Georgia Southern University, Statesboro, GA, United States; ^3^ Department of Mechanical Engineering, Georgia Southern University, Statesboro, GA, United States

**Keywords:** H-reflex, peripheral neuropathy, prone, standing, heel-contact

## Abstract

**Purpose:** The project was to examine the influence of peripheral neuropathy (PN) severity on the relationship between Hoffmann-reflex (H-reflex) and postures.

**Methods:** A total of 34 participants were recruited. H-reflex (H/M ratio and H-index) during prone, standing, and the heel-contact phase of walking was tested, along with foot sole sensitivity.

**Results:** The participants were divided into three groups based on the severity of the foot sole sensitivity deficit: control, less (LA), and more (MA) affected with both feet 5.07 monofilament test scores ranging 10, 0–5, and 6–9, respectively. A significant group by the posture interaction was observed in the H/M ratio (F_3.0, 41.9_ = 2.904, *p* = 0.046, *η*_*p*_^*2*^ = 0.172). In the control group, the H/M ratio of prone (22 ± 7%) was greater than that of the standing (13 ± 3%, *p* = 0.013) and heel-contact phase (10 ± 2%, *p* = 0.004). In the MA group, the H/M ratio of standing (13 ± 3%) was greater than that of the heel-contact phase (8 ± 2%, *p* = 0.011). The H-index was significantly different among groups (F_2,28_ = 5.711, *p* = 0.008, and *η*_p_^*2*^ *=* 0.290). *Post hoc* analysis showed that the H-index of the control group (80.6 ± 11.3) was greater than that of the LA (69.8 ± 12.1, *p* = 0.021) and MA groups (62.0 ± 10.6, *p* = 0.003).

**Conclusion:** In a non-PN population, the plantar sensory input plays an important role in maintaining standing postural control, while as for the PN population with foot sole sensitivity deficiency, type Ⅰ afferent fibers reflex loop (H-reflex) contributes more to the standing postural control. The H-index parameter is an excellent method to recognize the people with and without PN but not to distinguish the severity of PN with impaired foot sole sensitivity.

## Introduction

Peripheral neuropathy (PN) is a neurodegenerative disease caused by peripheral nerve damage, with up to 20% in the elderly (Richardson, 2002). It gradually affects the peripheral nervous system (PNS) from the tips of the limbs to the spinal cord (Li et al., 2019). The main symptoms are impaired nerve conduction and abnormal sensation (pain and numbness) starting at the foot sole (Hanewinckel et al., 2016). Peripheral nerve damage may occur in sensory afferent and motor nerve fibers (Magrinelli et al., 2015). The damage to sensory afferent fiber can lead to the foot plantar sensitivity deficit (Bernard-Demanze et al., 2009). The affected motor nerve fiber causes abnormal performance in the lower limb. PN could lead to decreased nerve conduction velocity (NCV) and the type Ⅱ afferent pathway that innervates the receptors under the skin (Zhang et al., 2015b). The impairment of the nervous system changes the neuromodulation pattern of people with PN to maintain the body’s posture stability and functional gait compared to the non-PN population (Li et al., 2019).

Hoffmann-reflex (H-reflex), a surrogate measure of the stretch reflex, has been an effective method for testing neuromodulatory processes for healthy and pathological populations (Wager and Buerger, 1974). The neural pathway of the stretch reflex arc connects type Ia afferent fibers of the muscle spindle to the α-motoneurons, which are involved in balance and posture control (Andersen and Sinkjaer, 1999). The H-reflex test produces two outcome parameters. The H/M ratio is used to test the excitability of α-motoneurons (Pierrot-Deseilligny and Mazevet, 2000), which are affected by presynaptic inhibition. The H-index is used to test NCV in the reflex pathway (Guiheneuc, 1971).

Postures influence the testing results of H-reflex among the healthy population of different ages (Hayashi et al., 1992; Ethier et al., 2003). The lower limb muscles are relaxed, and there were minimal excitatory or inhibitory adjustments for α-motoneurons during lying in the prone position. The lower limb muscle activation increased for postural control during standing, where the central nervous system (CNS) suppresses the reflex activity in the young population. The H/M ratio tested in the prone position was proved to be greater than that in standing in young adults, although the opposite trends were reported in the elderly population (Angulokinzler et al., 1998). The H-reflex of standing was also reported to be different from that of walking (Capaday and Stein, 1986), where the magnitude of H-reflex was greater during maintained contractions (standing) than during walking. The difference was the most significant during low-level activity, such as the heel-contact phase of walking. The level of CNS inhibition increased from prone to standing and then increased again to walking.

Several studies reported age-associated increases in muscle coactivation during postural control (Wu, 1998; Allum et al., 2002; Tucker et al., 2008). The CNS enhances its regulation of the excitation of the PNS with aging. Compared with the younger population, the gastrocnemius muscle activity level of the elderly population was greater during the loading response, and the lower limb muscle coactivation increased during walking (Schmitz et al., 2009). The elderly population showed a smaller H-wave than younger populations. (Capaday and Stein, 1986; Chalmers and Knutzen, 2000; Knikou et al., 2011; Raffalt et al., 2015).

It was also reported that the H-reflex parameters were different in different pathological populations. The H-reflex latency was prolonged when the H-reflex amplitude was reduced following hip flexion, and hip extension shortened the reflex latency in the spinal cord-injured patients (Knikou and Rymer, 2002). Due to impaired foot sole sensitivity among people with PN, type I afferent contribution increases, and stretch reflex plays an important role in maintaining posture (Zhang et al., 2015b). Moreover, the lower limb muscle atrophy among people with PN caused changes in the muscle activity and coactivity levels. Research has shown that H-reflex (H/M ratio and H-index) was an effective tool for the investigation of the effects of PN on movement control (Guiheneuc and Bathien, 1976). Kneis et al. (2016) focused on the chemotherapy-induced peripheral neuropathy (CIPN) study. They observed that CIPN prolonged H-wave latency and decreased H-reflex elicitability during standing compared with healthy controls.

The soleus (SOL) has proved reliable in H-reflex testing in healthy young adults (Handcock et al., 2001; Simonsen and Dyhre-Poulsen, 2011), older adults (Mynark, 2005), and the population with spinal cord injury (Phadke et al., 2010). However, it was reported that SOL function declines faster with aging than gastrocnemius (Ebrahimi et al., 2020). The lateral gastrocnemius was proved to be the most reliable muscle in prone (Zhang et al., 2015b), standing, and walking (Song et al., 2022) for the H-reflex tests in people with PN. The LG is a better choice for H-reflex tests in the PN population.

The balance of the non-PN population is mainly maintained by tactile feedback of the foot plantar transmitted by the type II afferent fibers. In people with PN, the type II afferent pathway is damaged due to impaired plantar sensitivity, leading to be more dependent on the type I afferent pathway to maintain balance (Li et al., 2019). Therefore, the H-reflex modulation may differ in different postures with and without PN. Few studied the effects of the severity of PN on the H-reflex modulation with postures, although many investigated the effects of disease on H-reflex. However, further understanding of the effects of PN on H-reflex could be valuable for the rehabilitation and treatment of people with PN and lay the foundation for the further exploration of the disease mechanisms.

We aimed to examine the influence of PN severity on the relationship between H-reflex and different postures in this project. We hypothesized that the H/M ratio adaptation to postures would change from the non-PN to PN population, and the adaptation changes with the severity of PN. We further hypothesized that the severity of PN would negatively affect the H-index in all postures compared with the population without PN.

## Methods

### Experiment Procedure

A total of 38 participants were recruited, including the non-PN and PN population. The PN population was recruited from the local community based on doctor-diagnosed peripheral neuropathy (PN) with varying degrees of foot sole sensitivity. The contraindications that cannot be controlled with medications or other physician-prescribed therapies and affect daily exercise ability were excluded from this study: 1) heart condition; 2) high blood pressure; 3) spinal cord disease; 4) losing balance because of dizziness or lost consciousness within the past 12 months; 5) bone, joint, or soft tissue problem that could be made worse by becoming more physically active; and 6) physical activity need to be medically supervised. The inclusion criteria were evidence of PN symptoms, including pain and numbness at the bottom of their feet, aged 65 or older. The local Institutional Review Board approved this research project (H20076). All the participants signed the informed consent before data collection. Bilateral foot sole sensitivity was tested using monofilaments (Feng et al., 2009). H-reflex was then evoked by stimulating the tibial nerve in the prone, standing, and heel-contact phase of walking conditions on the right leg. Reliability of H-reflex tests using SOL muscle in prone (Zhang et al., 2015a), standing, and walking (Song et al., 2022) within people with PN have been documented.

### Plantar Pressure Sensitivity Test

Foot sole sensitivity was assessed with a 5.07-gauge Semmes–Weinstein monofilament (North Coast Medical, Inc., Morgan Hill, CA, United States). Five locations of each foot, hallux, bases of first/fifth metatarsals, mid-sole, and heel were assessed (Nurse and Nigg, 2001). Each site was tested three times in a random order. The detailed foot sole sensitivity testing protocol can be found in our earlier work (Li and Manor, 2010).

### H-Reflex Measurements

The participants warmed up on a treadmill (AMTI, Watertown, MA, United States) with a self-selected speed for 5 min or until they felt comfortable walking on the treadmill. We located the optimum site of nerve stimulation with a hand-held electrode using the criterion that Ia afferents could be selectively stimulated at low stimulus intensities (Ferris et al., 2002). A pre-gelled disposable 2 × 2 cm cathode replaced the hand-held electrode at the selected site in the popliteal fossa. Also, a 5 × 8 cm anode was placed over the patella. The reaction to the stimulation was collected using surface electromyography (EMG) electrodes (Trigno Wireless EMG System; Delsys Inc., Massachusetts, United States) attached to the belly of the right lateral gastrocnemius muscle. We selected the right side to test to compare with the literature (Crenna and Frigo, 1987; Chalmers and Knutzen, 2000; Raffalt et al., 2015) since there was no evidence for the difference between the left and right sides. The LG was selected rather than the traditional soleus muscle since the H-reflex of LG was more reliable than that of the soleus in prone (Zhang et al., 2015b), standing, and walking (Song et al., 2022) for people with PN.

**FIGURE 1:**
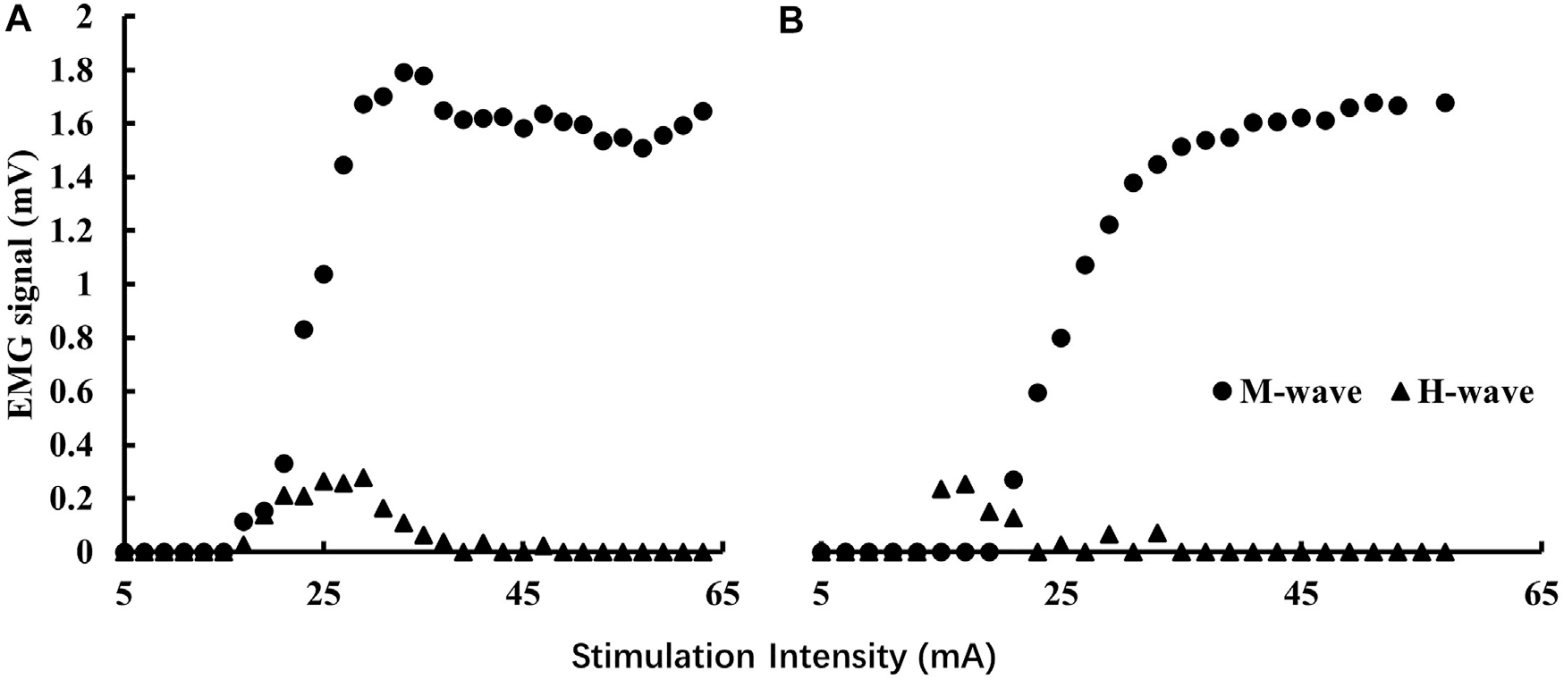
Exemplary H-reflex recruitment curves for prone **(A)** and standing **(B)** conditions. The magnitudes of the background EMG activities were significantly less than those of the M-wave so they are not visible in the graphs.

Prone H-reflex was tested with the participants lying prone on an examination table and keeping the spine straight with arms on both sides of the body. The H-reflex was elicited by a 500 μs square-pulse constant current stimulus (Digitimer model DS7A, Digitimer Ltd., Welwyn Garden City, England) (Chen and Zhou, 2011). We chose the low end of the recommended 0.5–1 ms square wave width (Panizza, et al., 1989) to avoid potential discomfort experienced by the participants. Stimulation started from 5 mA and increased with 2 mA to achieve the full H-reflex recruitment curve at an inter-stimulus interval of 10 s (Laudani et al., 2009). Standing H-reflex was then tested with the participants standing with their feet apart at a shoulder width and the ankles at a neutral position. Arms were relaxed on the side of the body. Eyes looked straight ahead and kept the spine neutral. The same procedure as the prone position was used to obtain the recruitment curve. The exemplary recruitment curves for prone and standing conditions are shown in Figure 1.

The treadmill was used for the walking H-reflex test for the following reasons: the participants were equipped with safety belts during walking on the treadmill to ensure safety during testing. Most literature used the treadmill for the H-reflex test while walking (Capaday and Stein, 1986; Ethier et al., 2003; Larsen et al., 2006). Then, the treadmill can ensure a consistent gait cycle for stimulation accuracy while walking.

A customer-made electrical footswitch, taped under the right heel of the shoe, was used to detect heel contact during walking. The footswitch was connected to a LabVIEW board, and the LabVIEW board was connected to the stimulator. Two consecutive heel contacts defined a stride cycle. Eleven consecutive heel contacts were collected when walking at a self-selected speed. Average stride cycle durations were calculated from the raw data of ten stride cycles. Based on the stride cycle duration results, a customized LabVIEW program triggered the stimulator at 5% of the stride cycle, defined as heel-contact (Krauss and Misiaszek, 2007; Querry, 2008). Multiple H-reflexes were evoked to ensure 10 quality trials. Stimulus intensity was set to the equivalent intensity that produced a 15% maximum M-wave during the standing condition (Raffalt et al., 2015). The stimuli were given at least 2s apart from one another (Raffalt et al., 2015).

The testing order was fixed as prone, standing, and walking, to avoid the effect of muscle activity on the results of the less active testing conditions, for e.g., the effects of standing on prone, and the effects of walking on prone and standing.

### Data Processing

**FIGURE 2:**
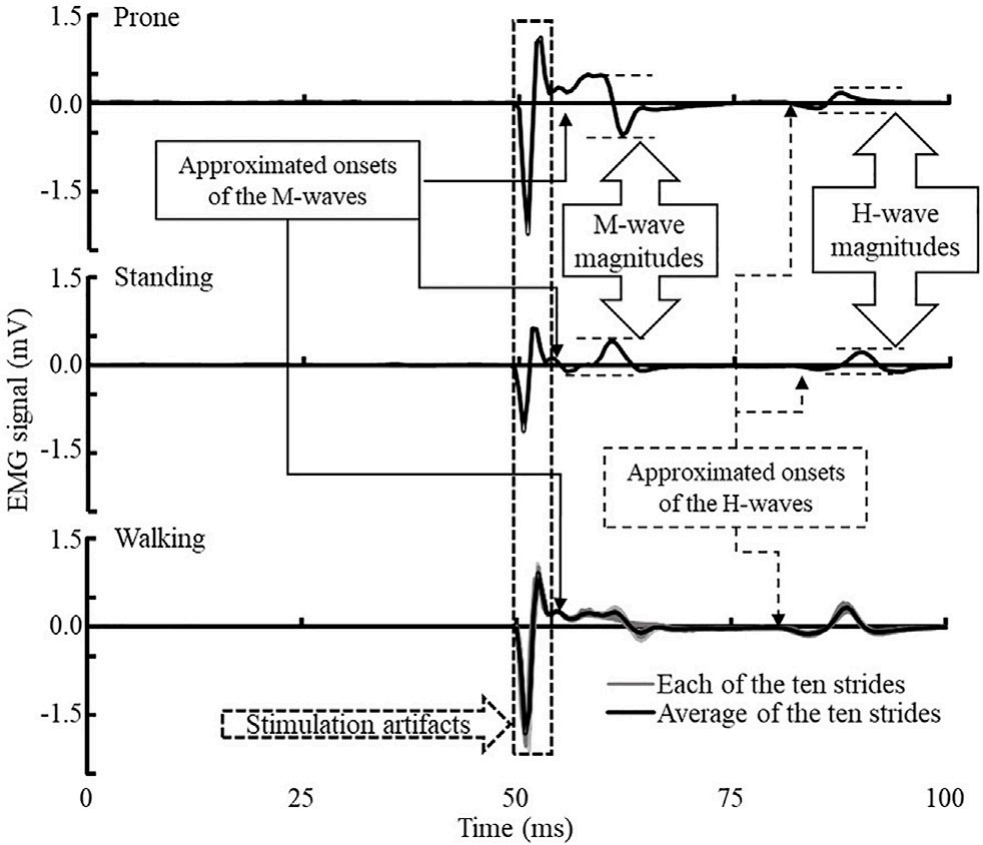
Exemplar H-reflex EMG data of prone (upper), standing (middle), and walking heel-contact (lower) conditions. The onsets of H- and M-wave were the first deflection after a short stabilized period of the EMG signals. Walking EMG collection was triggered by the footswitch at foot contact, and no background EMG was collected before the H-reflex signals. Background EMG signals were negligible compared to the magnitudes of the M- and H-waves during all three conditions.

All EMG data were collected using a sampling rate of 2000 Hz with the embedded band-pass filters of 20–450 Hz in the Delsys electrodes. EMG data were processed using Delsys software. The magnitudes of H-wave and M-wave were calculated with the peak-to-peak amplitude of the corresponding signals. The H/M ratio of prone and standing was the maximum of H-wave (Hmax) divided by the maximum of M-wave (Mmax). However, the H-wave of walking was expressed as an average of over ten measurements in the heel-contact phase. The H/M ratio of heel-contact was calculated as H-wave divided by the standing Mmax. The H-index is the relative onset latency of the H- and M-waves. Figure 2 presents more detailed information regarding identifying the relevant outcome variables. Note that background EMG in all three conditions was negligible among the participants of this project. No treatment of background EMG signals was employed in this project since they were not interfering with our identification of the outcome variables. The H-index is calculated using the following equation (Scaglioni et al., 2002):

$$H-index\;=\;{\left[\frac{Height\left(cm\right)}{\Delta t_{H}-\Delta t_{M}}\right]}^{2}*2,$$

where Height (cm) refers to the height of the participant and $\Delta t_{H}-\Delta t_{M}$ represents the latency between the onsets of H-wave and M-wave.

The interval times of the maximum H-wave and M-responses and participants’ height were used to calculate the H-index under the prone and standing conditions. The H-index during the heel-contact was calculated by the H-wave and M-response of the average value of the ten tested curves.

### Statistical Analysis

Statistical analysis was performed using SPSS statistics 24.0 (SPSS Inc., Chicago, IL). One-way ANOVA examined potential group differences in age, body mass (Chen, #51), height, and body mass index (BMI). The differences in H-reflex parameters (H/M ratio and H-index) were examined using a two-factor (posture by the group) mixed-model ANCOVA with BMI as a covariant (BMI is a potential confounding factor; see Fujimaki et al., 2009 for more details). The effect size of the ANCOVA is represented by *η*_p_^*2*^. Small, medium, and large effect sizes are defined as *η*_p_^*2*^ <0.01, 0.01≤*η*_p_^*2*^ <0.06, and 0.06≥*η*_p_^*2*^, respectively (Cohen, 1988). Bonferroni pairwise comparison was used as a *post hoc* test wherever appropriate. Cohen’s *d* was used to evaluate the related effect size. Small, medium, and large effect sizes are represented by 0.20≤d<0.50 , 0.50≤d<0.80 , and d≥0.80 , respectively (Cohen, 1992).

## Results

A total of 38 people with PN were recruited for this study. Four participants were excluded from the analysis due to the inability to evoke H-reflex. Participants were separated into the control, less (LA), and more (MA) affected groups based on our observed foot sole sensitivity. The participants in the control group had full foot sole sensitivity-10 scores. The participants in the LA group had better plantar pressure sensitivity scores, ranging from 6 to 9. Participants in the MA group had lesser (0–5) plantar pressure sensitivity scores. The benefit of this grouping was dividing the participants into three groups based on the severity of the disease. It enabled us to discuss the effects of PN with different levels of foot sole sensitivity on different postures.

Normality of the results was tested using the Shapiro–Wilk test, and all of the H/M ration and H-index results were normally distributed; therefore, parametric analysis was used as results.

### Participants

The demographic characteristics of the three groups are presented in Table 1. There was no difference observed in age. The body mass and height of MA were significantly greater than those of the control and LA groups. The BMI of the MA group was significantly greater than that of the control.

**TABLE 1:** Demographics and functional mobility variables for three groups.

	Control (*n* = 9)	LA (*n* = 13)	MA (*n* = 12)	F_2,33_	*p-*value
	Mean ± SD	Mean ± SD	Mean ± SD		
Age (yr)	70.9 ± 3.6	74.9 ± 5.5	73.6 ± 5.2	1.788	0.184
Body mass (kg)	69.6^a^ ± 11.8	75.7^a^ ± 14.4	95.9^b^ ± 24.2	6.484	0.004*
Height (cm)	163.3^a^ ± 6.2	164.1^a^ ± 8.1	174.0^b^ ± 9.7	5.975	0.006*
BMI (kg/m^2^)	26.0^a^ ± 4.0	28.1 ± 4.5	31.3^b^ ± 5.4	3.332	0.049*

Note: BMI, body mass index; * indicates statistically significant difference among groups as results of the ANOVA; ^a,b^ indicate homogenous groups, different letters represent groups that are significantly different from each other as results of the *post hoc* pairwise comparison.

### H/M Ratio

**FIGURE 3:**
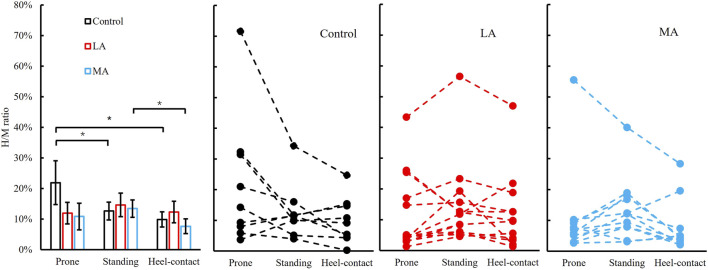
Left panel: H/M ratio (Mean ± SEM) of three postures (prone-solid line, standing-dash line, and heel-contact-dotted line) among three groups (control, LA, and MA). * indicates a statistically significant difference among postures in the same group. Right three panels: individual data points in control, LA, and MA groups are presented for all three postures.

One participant of the MA group could evoke H-reflex in the prone position, and one more person failed to get H-reflex in the heel-contact phase. In the LA group, one participant failed to evoke the H-reflex in the heel-contact phase. We observed a significant group by the posture interaction in the H/M ratio (F_3.0, 41.9_ = 2.904, *p* = 0.046, and *η*_*p*_^*2*^ = 0.172). The results of the *post hoc* analysis were as follows: in the control group, we observed that the H/M ratio of prone (22 ± 7%) was greater than that of the standing (13 ± 3%, *p* = 0.013, and *d* = 0.566) and heel-contact phase (10 ± 2%, *p* = 0.004, and *d* = 0.749). In the MA group, the H/M ratio of standing (13 ± 3%) was greater than that of the heel-contact phase (8 ± 2%, *p* = 0.011, and *d* = 0.630). Figure 3 shows more details and the raw data for all three groups across all three conditions.

### H-Index

**FIGURE 4:**
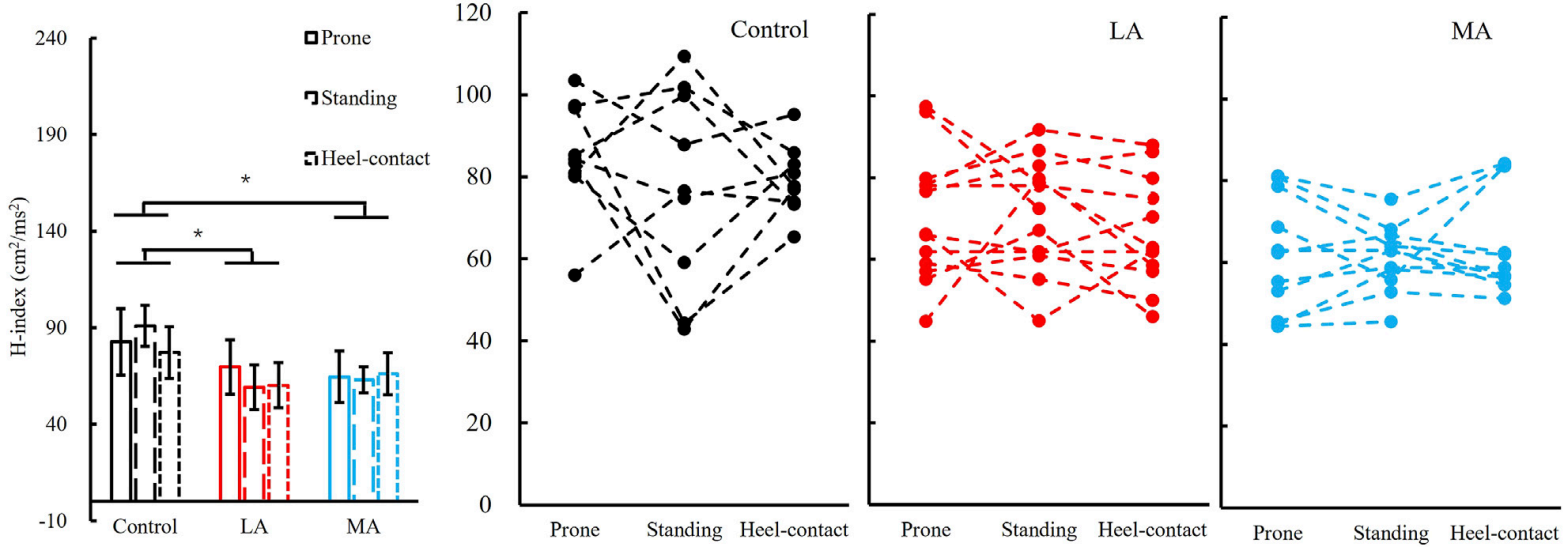
Left panel: H-index (Mean ± SEM) of three groups (control, LA, and MA) with all postures (prone, standing, and heel-contact) combined since there was no posture difference detected. * indicates a significant pairwise difference between two postures. Right three panels: individual data points in the control, LA, and MA groups are presented for all three postures.

We failed to observe a significant group by the posture interaction in the H-index. The H-index was significantly different among groups (F_2,28_ = 5.711, *p* = 0.008, and *η*_p_^*2*^ *=* 0.290). *Post hoc* analysis showed that the H-index of the control group (80.6 ± 11.3) was greater than that of LA (69.8 ± 12.1, *p* = 0.021, and *d* = 0.916) and MA groups (62.0 ± 10.6, *p* = 0.003, and *d* = 1.709). More details are shown in Figure 4.

## Discussion

We have set out to check the hypotheses that the differential influence of posture on H-reflex parameters among people with different severity of foot sole sensitivity in PN. Significant differences were observed in the H/M ratio among postures within groups. Postures affected the H/M ratio more in the control than that in LA and MA groups, which fully supports our first hypothesis. More specifically, the significant decrease of the H/M ratio from the prone to the standing and heel-contact phase in the control group was not observed in the other two groups. Meanwhile, the standing H/M ratio was much greater than the h-contact H/M ratio in the MA group. Significant differences were observed in the H-index among groups. The H-index in the control group was much greater than that of LA and MA groups, which is consistent with our second hypothesis.

In our study, 89% of participants successfully elicited the H-reflex, which was consistent with the 79% (Scaglioni et al., 2002) to 90% (Chen et al., 2015) successful rate in the literature. There are a few potential reasons for failing to elicit H-reflex. One of those four participants had a spinal injury. The related nerves may be damaged, and the nerve conduction pathway was impaired; the other three may have been affected by aging-related functional decline or PN disease. Serious PN causes further damage to axons and myelin of peripheral nerves (Li et al., 2019), leading to failure to evoke H-reflex; another possible reason was the antidromic wave along the motor nerve eliminated the descending H-wave (see Knikou, 2008, for more details).

The effects of PN symptoms severity on the H/M ratio have not previously been reported. We observed significant differences between prone, and standing and walking conditions within the control group but not within the LA and MA groups. These results are consistent with previous research on the elderly population. A large number of researchers (Koceja et al., 1995; Angulokinzler et al., 1998; Kim et al., 2013; Raffalt et al., 2015; Cecen et al., 2018) observed that the H/M ratio decreased from prone to standing for young participants but increased from prone to standing in the elderly participants. The differences we observed between the standing and heel-contact during walking are also consistent with previous research. The H/M ratio of the heel-contact phase of gait among the MA group was suppressed, the same as the other research on the healthy population (Chalmers and Knutzen, 2000; Krauss and Misiaszek, 2007). The critical observations here were that the severity of foot sole sensitivity loss significantly affects the H-reflex magnitude across all three conditions.

The H-index between control and LA/MA groups differed but not among postures. The latency of the H-reflex indicates the efficiency of synaptic transmission between the afferent and α-motoneurons, including the conduction velocity of both efferent and afferent nerves. The results of the H-index among the control, LA, and MA groups are consistent with the literature (Guiheneuc and Bathien, 1976). They reported that the H-index was an effective parameter for differentiating PN from healthy populations. Also, Zhang et al. (2015b) observed that the H-index of people with PN was less than that of the healthy population in the prone position. Same as these reports, we observed that the H-index of the control group was greater than that of the other groups with more severe foot sole sensitivity impairment. However, the difference was not observed between the LA and MA groups.

The H/M ratio showed important information in adaptation to postures within the population with postural control and foot sole sensitivity deficiency. The H-reflex magnitude of the three postures was different in the three groups. There was a difference between prone and standing positions in our study for both outcome variables in the control group. The insensitivity of H-reflex from prone to standing was observed in the other two foot sole sensitivity deficiency groups. Foot cutaneous sensation afferents play an important role in standing postural control among healthy populations (Chen et al., 1995; McKeon and Hertel, 2007; Zhang and Li, 2013; Zhang et al., 2015b). However, due to the degeneration in the foot cutaneous sensation, people with PN may rely more on large diameter sensory fiber afferents to maintain balance when standing compared with the healthy population (Chen et al., 1995; McKeon and Hertel, 2007; Zhang and Li, 2013; Zhang et al., 2015b). As a result, compared with the prone position, presynaptic inhibition could be increased, the spinal reflex could be declined, and the H-reflex magnitude is decreased during standing among the healthy population. Still, the PN population with foot sole sensitivity deficiency is less adaptable to this change.

Furthermore, the H/M ratio was relatively low during the heel-contact phase in the walking compared to prone or standing positions, which was consistent with other research (Yang and Whelan, 1993; Ethier et al., 2003; Knikou et al., 2011). The pathology did not change the reflexive behavior at the beginning of the stance phase. In our study, the cutaneous sensation of the PN population was damaged, which decreased the cutaneous afferents in the walking. Cecen et al. (2018) suggested that the lower extremity proprioceptors may presynaptically interfere with the effectiveness of the spindle primary afferent synapses on the soleus motor neurons. Extending this hypothesis to our observations, we like to propose the following: the non-significant reduction of the H/M ratio from prone to standing reflects reduced adaptability in neural modulation among the PN population with impaired foot sole sensitivity. Significant reduction observed during the heel-contact phase to standing indicates more significant proprioceptive interference during walking in this population. This interference had a greater influence on the spindle primary afferent synapses of the tibial nerve, leading to the reduction of the H/M ratio.

The H-index of the control group showed greater NCV than that of the LA and MA groups, which is consistent with the literature (Guiheneuc and Bathien, 1976; Zhang et al., 2015b). Guiheneuc and Bathien, (1976) observed that the H-index reduced indicated peripheral NCV decreased in the PN population with uremic and alcoholic compared with the healthy population. Zhang et al. (2015b) reported that the H-index of PN with impaired plantar sensitivity was less than that of the age-matched population. In our study, the H-index was not affected by postures and is only associated with the severity of PN disease. It indicates that impaired sensory inputs are a major feature of PN with impaired plantar sensitivity distinguished from the healthy population.

It is possible to take advantage of the adaptation to the severity of PN observed here in training or rehabilitation programs. It was reported (Navarro et al., 2007) that the functional deficits of nerve injuries can be compensated by regeneration of injured axons or collateral branching of undamaged axons in the vicinity, and the remodeling of nervous system circuitry is related to the lost functions. In the case of the PN population, Li and Manor, (2010) observed that foot plantar sensitivity and posture stability could be improved through the 24-week Tai Chi exercise. Hongwei Guan, (2011) compared the two populations with and without Tai Chi training on H-reflex characteristics. They observed Tai Chi trainers demonstrated more inhibition in the motor pool in the standing position. That result indicated that Tai Chi exercise might affect the spinal inhibition system, influencing motor excitability. The immediate cause might be that the muscle reflex reaction adaptation was improved after long-time Tai Chi (Trimble and Koceja, 2001; Fong and Ng, 2006). The plasticity of the nervous systems can help when designing rehabilitation programs for people with PN.

Several important limitations need to be considered. First, we did not separate male and female participants in our analysis. Future studies might include potential differences between male and female participants. Considering the safety of the older adults with PN during walking, we did not complete the recruitment curve during walking. The stimulation level at 15% Mmax of standing produced a consistent level of Hmax during walking. We have also used 2s stimulation intervals during walking to avoid fatigue potential. The result showed that our testing method during walking was safe and effective. The optimal level of stimulation and testing duration during walking can be explored in the future if fatigue can be avoided and safety can be maintained. The testing order of three postures (prone, standing, and walking) was not random and may have affected the results. We have taken some measures to minimize the impact, such as maintaining the participants’ psychological state and body temperature. The effect of the test order on H-reflex needs further study.

In conclusion, the H-index parameter is an excellent method for distinguishing between people with and without PN but is not sensitive to detect the severity of the PN-induced foot sole insensitivity. For people with a slight lack of foot sole sensitivity, the modulation of H-reflex is reduced, and type Ⅰ afferent fibers compensate for slightly impaired foot sole sensitivity in prone, standing, and walking. Compared with people with less foot sole sensitivity deficiency, type I afferent fiber reflex loop (H-reflex) contributes more to standing postural control to compensate for foot sole numbness in people with severely impaired foot sole sensitivity. This adaptive change could help people with PN reduce the risk of falls. The appropriate training to take advantage of this adaption could facilitate the regeneration of peripheral control, thereby restoring postural control.

## Data Availability

The original contributions presented in the study are included in the article/Supplementary Material; further inquiries can be directed to the corresponding author.
